# The Formation of Coagula in the Circulatory System

**Published:** 1861-04

**Authors:** E. L. Holmes

**Affiliations:** Chicago


### THE FORMATION OF COAGULA IN THE CIRCULATORY SYSTEM.

BY E. L. HOLMES, M. D., of Chicago.

(*Read before the Chicago Medical Society.*)

Few pathological changes offer a more interesting subject of study than the formation of clots in the veins and arteries. Although in many of our best standard works upon Pathology and "The Practice of Medicine" there is scarcely more than an allusion to the subject, yet it has for more than two hundred years excited the interest of accurate observers, some of the most distinguished of whom in our time have devoted much labor to its investigation and have contributed valuable additions to the literature of our science. Several important facts have been established, by which curious phenomena in the progress of certain diseases can be satisfactorily explained. It is true the cases in which several of these phenomena are observed, are somewhat rare, yet they are liable to occur, and have without doubt occasionally fallen under the observation of every experienced practitioner. I regret that I have been able to consult so few original authorities and consequently cannot offer a paper so extended as I would wish or as the subject merits. I may mention the fact that I have found the *titles* of twenty-five articles relating more or less intimately to the subject, several of which are said to be monographs of great learning and research, prepared by writers whose authority stands prominent in medical science.

Virchow states that it had long been a popular idea that the blood under certain circumstances "curdles" in the veins during life; but that the attention of the profession was first drawn to the subject by Coiter, of Nuremburg, in 1572, and more particularly by Severinus in 1616. For two centuries physicians looked upon the fibrinous collections, found in the heart and larger vessels, as the cause of numerous diseases, especially of the lungs. In more recent times it had become almost universally the opinion that in all cases these fibrinous masses were formed after death. Within a few years, however, the subject has been carefully studied by pathologists, and the fact has been placed, perhaps beyond a doubt, that coagula may form in the heart and in different parts of the venous and arterial systems, and be the cause primarily or secondarily of the most grave consequences.

These coagula may be conveniently divided into three classes: 1st. Those originating in the heart and found in its cavities after death. 2d. Those formed in the veins and arteries, and caused by disease of those vessels in the locality in which they are found. And 3d. Wandering coagula, formed in certain localities of the circulating system and carried by the current of the blood to positions more or less remote from their origin. It is to this latter class that I wish particularly to call your attention.

1st. That the blood may coagulate in the heart during life is a fact, we think, now well established by the observations of the most reliable pathologists. Numerous cases are reported of obscure affections of the heart, arising in diseases in which the blood is known to contain an abnormal amount of fibrin. Subsequent examinations have revealed very firm yellowish concretions closely attached to the columnæ and valves, and presenting an appearance very different from that of the clots usually found after death in the heart and large vessels. That fibrin may collect in firm clot around the columnæ and valves is clearly shown by the facts not only that even in apparent healthy condition of the blood, the cardiac valves are sometimes found with quite large fibrinous concretions attached to them, but also that threads and other foreign substances, passed transversely through arteries of living animals, are soon covered with fibrinous concretions of considerable length. We see no reason why, in certain abnormal conditions of the blood, favoring these concretions, they may not form and attain a remarkable size.

For more extended discussions of this part of the subject, we would refer to the November number of the *London Lancet* for 1851, and to the January, March and October numbers of the same journal for 1852; also to page 404, vol XXII, of the *American Medical Journal.*

Somewhat different from what occurs in these cases is the more sudden coagulation of the blood in the cardiac cavities of women who have suffered from dangerous flooding in labor. If the blood taken from the vessels under such circumstances coagulates, as is said, with great rapidity, there is nothing improbable in the supposition that it may coagulate suddenly in the heart when brought almost to complete stasis, as it is in some cases of flooding from want of nervous power. It is well known that an attempt to raise such patients to an erect position is sometimes attended with fatal syncope. In other cases, the current of blood is not so much diminished, that stimulants may not arouse the patient after death has seemed inevitable. Cases are reported in which such patients, on being aroused, have manifested alarming symptoms of disease of the heart, which have terminated fatally at periods varying from two to eighteen days. No symptoms of cardiac disease had been previously observed and the autopsies revealed no abnormal appearances except the presence of firm clots, such as I have mentioned. The subject is discussed more fully in a Monograph by Prof. Meigs and alluded to in his work on Obstetrics, as also by Prof. Flint in his work on the heart.

2d. Coagula formed in the veins and arteries caused by disease of these vessels in the locality in which the coagula are found.

The pathology of the diseases of the arteries and veins is so well understood and is so extensively discussed in the best works upon Pathology, that I shall say but little upon this portion of the subject. Among the abnormal conditions, which primarily or secondarily produce obstruction, and often, in consequence, coagula in the veins and arteries, may be mentioned: 1st. Pressure caused by tumors, and the deposition of lymph and tuberculous matter in the tissues near the vessels; pressure caused by the gravid uterus, and by contractions produced by the cicatrization of diseased tissues involving an artery or vein. 2d. Enlargement of the vessels, as in case of aneurism, varices, and dilatation of the auricles of the heart, all which are known at times to contain fibrinous collections. 3d. Debility of the heart and inactivity of the capillaries, especially in old age and certain depressing diseases, when the blood gradually ceases to flow in the vessels. 4th. Local inflammation of the veins or arteries, which often, if extensive or not arrested by treatment, terminates in obstruction of the vessels. 5th. Atheromatous degeneration of the coats of the arteries, which roughen the lining membrane and often diminishes the inner diametre of the artery. Fibrinous collections are often deposited upon these roughened portions of the artery and sometimes entirely obstruct the current of blood. I have witnessed six autopsies, in which the basilar artery of the brain was either much roughened from atheromatous deposit or nearly obstructed from the same cause. Numerous cases are reported in which serious consequences have been traced to some one of the various diseased conditions just enumerated.

3d. Wandering coagula. Several Pathologists of eminence have made wandering clots the subject of special study. During the past few years interesting papers have been published by Dubini, Paget, Tufnell, Cruveilhier, Kirkes and Virchow. The latter Pathologist has probably investigated the subject more fully and has made more numerous examinations of the veins and arteries in the dead subject than any other writer. In an elaborate article of great interest in the "Hand Book of Special Pathology and Therapeutics," edited by Virchow himself, he has presented a very extended review of the subject. The principal facts connected with the phenomena of wandering clots may be thus briefly stated: Fibrin is deposited on the edges of the valves of the heart till concretions of considerable size are formed. These, from subsequent softening, or from the constant motion of the valves, may be dislodged from their seat and carried by the arterial current to a remote vessel small enough to arrest the course of the moving clot. The same thing may happen in cases of coagula formed upon the irregularities produced by atheromatous degeneration in the walls of an artery. Clots are also formed behind the valves of the veins, as in certain cases of varix, become dislodged and carried to the heart, and finally left in some artery of the lungs too small to allow its further progress.

Coagula formed locally in the vessels in any of the five ways above described may be broken into fragments, by pressure produced by sudden motion of a limb or by external violence, and, thus detached from their primary position, may be carried forward to obstruct the circulation of distant vessels.

It is evident that wandering clots formed in the veins must usually be arrested in the lungs; those originating in the left portion of the heart will be carried to some portion of the general arterial system; while those forming in the veins of the chylopoietic vicera must pass into some of the branches of the portal vein. Virchow has given the term Emboli to these wandering clots.

That the current of the blood is sufficiently strong to carry peices of coagula with it, is proved by the experiments of observers, who have injected small pieces of muscle, cork, India rubber and even metallic mercury into the vessels of animals, and have subsequently found them remote from the point of injection. It is not on theoretical grounds alone that wandering clots are stated to be a secondary cause of disease. Minute examination of the arteries and veins after death have disclosed coagulated fibrin completely filling the vessel for a short distance. The obstructing clots must have been formed elsewhere, since the artery presented no indication of disease either at the point of obstruction or in any other part. In very many of these cases disease of the heart with valvular excrescences have been observed, which resemble in all respects the coagula in the remote vessels. Moreover, cases are reported by Virchow, Kirkes, Bennet and others, in which serious symptoms of disease of various organs have suddenly appeared in certain valvular affections of the heart.

Prof. Bennet states that there are now many cases on record where, in conjunction with such diseases of the heart, clots have been found in the arteries leading to important organs, causing cerebritis, pneumonia, nephritis and splenitis.

According to Virchow, affections arising from the obliteration of an important artery from an Embolus differ from those produced by local diseases of the artery in the suddenness with which the symptoms appear.

There is scarcely need of dwelling upon the serious consequences, which must result from a sudden and extensive diminution of blood supplied to the brain, any of the viscera or extremities. Insanity, convulsions, atrophy, diminution of force, œdema, gangrene, and even death may follow. Coagula have been found in the coronary arteries of the heart, evidently originating from excrescences on the valves, where atrophy and insufficient nutrition were plainly the secondary cause of abnormal symptoms during life. It is stated on very high authority that gangrene of the extremities is often produced by the presence of wandering clots in the principal artery of the limb affected.

My attention was first directed to this subject some years since by remarks in reference to cases examined in the autopsy room of a very distinguished Pathologist. I have been led more recently to investigate the subject, from the fact that I was called upon to examine the left eye of a lady, which had become suddenly amaurotic four months before. The case may appear to have little connection with the question before us, and, I must confess, at the time I examined the eye, it did not occur to me that such a relation might possibly exist. On examining with the ophthalmoscope the eye affected, I found the transparent media perfectly normal. Externally also the eye seemed healthy, with the exception of a slight dilatation of the pupil. The posterior portion of the retina, instead of the yellowish red color peculiar to this part, as seen with the ophthalmoscope, was much darker than usual. The vessels of the retina were in a condition which I had never before observed, their course being marked only by dark opaque lines, much finer than the normal vessels. At the time I could not explain these abnormal appearances satisfactorily to myself, but gave my patient an unfavorable prognosis. It did not occur to me that it might be well to examine the heart, since in answer to my questions regarding her general health, nothing directed my thoughts to this point.

Two years after this, my attention was called to an interesting article, by V. Græfe, in the Archiv f. Ophthalmogie, vol. V., part I, page 136, devoted to the subject of emboli in the ophthalmic artery. He states that Virchow had already found peculiar clots in this artery in patients who had suffered from loss of vision. The description of Græfe's case illustrated by a plate of the vessels of the retina as seen by the ophthalmoscope, which so much resembled those of my own case, leads me to believe the two cases should be placed in the same class. In the case of Græfe, the suddenness of the attack, the absence of any apparent disease of the orbit, as tumor or infiltration of lymph, which could produce pressure upon the ophthalmic artery, seem to exclude all causes of the disease, except the presence of a small wandering coagulum. The examination of the heart by auscultation showed the existence of marked disease of the valves.

The subjects to which I have asked your attention are interesting ones and are worthy of a much more thorough investigation than I have been able to bestow. An elaborate monograph upon the subject would be a useful addition to our medical literature, and it is to be hoped that some one who has access to a sufficient number of original papers and reports will undertake such a work.

